# Effects of Replacing Fish Meal with Rubber Seed Cake on Growth, Digestive, Antioxidant and Protein Metabolism of Juvenile Asian Red-Tailed Catfish (*Hemibagrus wyckioides*)

**DOI:** 10.3390/ani14213149

**Published:** 2024-11-02

**Authors:** Tao Cheng, Han Huang, Haifeng Mi, Lu Zhang, Junming Deng, Shuang Zhang, Xiaohui Dong, Shuyan Chi, Qihui Yang, Hongyu Liu, Shiwei Xie, Wei Zhang, Beiping Tan

**Affiliations:** 1 Laboratory of Aquatic Nutrition and Feed, College of Fisheries, Guangdong Ocean University, Zhanjiang 524088, China; 15602477486@163.com (T.C.); zhangshuang198610@126.com (S.Z.); dongxiaohui2003@163.com (X.D.); chishuyan77@163.com (S.C.); qihuiyang03@163.com (Q.Y.); liu_hong_yu@163.com (H.L.); xswzsdx@163.com (S.X.); khzhangwei110@sina.com (W.Z.); bptan@126.com (B.T.); 2 College of Animal Science and Technology, Yunnan Agricultural University, Kunming 650201, China; tao153869@gmail.com; 3 Tongwei Agricultural Development Co., Ltd., Chengdu 610093, China; mihaifeng@tongwei.com

**Keywords:** rubber seed cake, *Hemibagrus wyckioides*, growth, antioxidant capacity, protein metabolism

## Abstract

An 8-week feeding trial was conducted to assess the impact of replacing fish meal (FM) with rubber seed cake (RSC) on growth performance, digestive enzyme activity of the gut, antioxidant capacity and protein metabolism-related parameters of plasma and liver in juvenile Asian red-tailed catfish (*Hemibagrus wyckioides*) with an average body weight of 3.21 g. Four isonitrogenous (41.08 ± 0.17) and isolipidic (10.07 ± 0.11) diets were formulated, incorporating RSC at a level of 0%, 15%, 30%, and 45% (*w*/*w*) to progressively replace FM. Dietary inclusion of 15–30% RSC did not significantly affect growth performance (*p* > 0.05). However, the replacement of FM with RSC resulted in linear reductions in intestinal trypsin and amylase activities, with a significant reduction in intestinal amylase activity observed at the 45% RSC inclusion level. Dietary inclusion of 30–45% RSC decreased plasma glutamate dehydrogenase and aspartate aminotransferase activities, but increased plasma adenosine monophosphate deaminase and hepatic γ-glutamyltransferase activities. This may indicate that the high substitution of FM by RSC may affect the homeostasis of amino acid metabolism. Dietary inclusion of 15% RSC did not affect serum and hepatic antioxidant enzyme activities; however, dietary inclusion of 45% RSC significantly increased plasma malondialdehyde level, but decreased catalase activity. Furthermore, dietary inclusion of 30–45% RSC significantly increased hepatic lactate dehydrogenase activity. These results demonstrated that replacing a portion of FM with RSC is feasible, and dietary inclusion of 15% RSC did not have significant adverse effects on growth performance, digestive function and antioxidant capacity of *H. wyckioides*.

## Simple Summary

In aquafeeds, due to the increasing price of fishmeal, much attention has been paid to research on the substitution of fishmeal by various plant proteins. The present experiment, on the other hand, was conducted to investigate the effects of rubber seed cake as a substitute for fishmeal on growth performance, antioxidant capacity, digestion and protein metabolism of Asian red-tailed catfish (*Hemibagrus wyckioides*). Ultimately, it was found that the addition of 15% RSC to the diet did not adversely affect the growth, digestive enzyme activities or antioxidant capacity of Asian red-tailed catfish. The results of this experimental study can guide the feed industry to develop more effective and scientific aquatic feeding strategies.

## 1. Introduction

The Asian red-tailed catfish (*H. wyckioides*) is an omnivorous freshwater fish that belongs to the order Siluriformes and the family Corydidae. This species is native to numerous rivers across several Asian countries, particularly in Thailand, Laos, Cambodia, Myanmar, and China [1]. *H. wyckioides* is distinguished by its diverse diet and its ability to thrive in a wide range of aquatic environments [2]. These characteristics render it an important species for extensive farming with significant commercial value. Despite its widespread cultivation in ponds and cages throughout the Lancang–Mekong River area, there is currently no specialized compound feed available for *H. wyckioides*. This absence is primarily due to the limited information regarding its nutrient requirements [3,4,5,6] and the utilization of feed ingredients [7,8].

According to the State of World Fisheries and Aquaculture, China’s aquaculture production constitutes 58.9% of the global total, establishing it as the leading producer worldwide [9]. The rapid advancement of intensive aquaculture has led to significant increases in the quantity, variety, and production of aquafeed [10], which has resulted in a marked rise in the demand for fish meal (FM) [11,12]. However, global FM production struggles to meet this escalating demand, leading to rising FM prices and a persistent imbalance between supply and demand in the market. This scenario adversely affects the development of the aquaculture industry [13]. To foster sustainable development within the industry, the aquatic feed sector urgently needs to identify alternative protein sources to diminish its reliance on FM. Among the various protein sources, plant protein emerges as a viable alternative to FM. In recent years, many studies have focused on exploring the replacement of fishmeal with plant proteins in aquaculture. Examples include the use of pea protein as a replacement for fishmeal in studies with rainbow trout [14]; and the use of beet and carrot leaf protein concentrates as a replacement for fishmeal in studies with tilapia [15]. Previous studies have indicated that plant protein sources can partially or completely replace FM under specific dietary conditions [16]. Nonetheless, traditional sources of plant protein, including soybean, cottonseed, and rapeseed meals, are becoming increasingly scarce, resulting in prices that exceed what feed manufactures can afford [17]. Consequently, to lower feed production costs and achieve sustainable aquaculture development, unconventional plant protein sources (particularly those derived from seeds, leaves, and other crop by-products) have become a focal point of research [18].

The rubber tree, native to South America, is a species of significant economic importance and is now cultivated across several nations in Asia, Africa, South America, and Oceania. The global area dedicated to rubber plantations spans 10,315,732 hectares [19], with an estimated annual production of rubber tree seeds reaching approximately 7,700,000 tons [20]. While the protein components of rubber tree latex have been extensively investigated, the protein present in rubber tree seeds has received comparatively less attention [21]. Rubber seed has high nutritional value: the crude protein content of fresh rubber seed is about 9%, the hydrocyanic acid content is about 0.2%, and the rubber hydrocarbon content is about 0.2%. Following oil extraction from the rubber seed kernel, the remaining cake contains 26.1% crude protein and 11.0% crude fat, 75.6 mg/kg cyanide, 31.5 g/kg phytic acid, and 5.2 g/kg tannin [22,23]. Therefore, to improve the utilization of feed resources and promote the sustainable development of aquaculture, it is essential to develop and utilize rubber tree seeds and their by-products as alternatives to FM. This study aimed to assess the effects of replacing FM with rubber seed cake (RSC) on the growth performance, digestive function, antioxidant capacity, and protein metabolism of *H. wyckioides*.

## 2. Materials and Methods

### 2.1. Experimental Diets

The primary protein sources utilized in this study were FM and RSC, while fish oil, soybean oil, and soybean lecithin served as the main fat sources. FM, containing 73.6% crude protein, was sourced from Kunming Tianyuan Feed Co., Ltd. (Yunnan, China). RSC, with crude protein content of 30.6%, was derived from the residue of decorticated rubber seed after oil extraction via mechanical pressing, provided by Xishuangbanna Huakun Biotechnology Co., Ltd. (Yunnan, China). The experimental feeds were formulated with varying proportions of RSC (0, 15, 30, and 45% (*w*/*w*)) to replace FM, resulting in the creation of four distinct diets (R0, R15, R30, and R45). All the diets were isonitrogenous (41% crude protein) and isoenergetic (21 MJ/kg gross energy). The ingredients and chemical composition of the experimental diets are detailed in Table 1.

Before formulating the feed, all the ingredients were crushed using a pulverizer (SFSP series; Kunming Huaming Grain, Oil and Feed Equipment Factory, Kunming, China), and subsequently passed through a 60-mesh sieve. The crushed feed ingredients were then mixed according to the formula outlined in Table 1. Following this, soybean oil and soybean lecithin (dissolve in soybean oil) were added, with the small grease particles being dispersed manually. The mixture was combined in a plastic film bag, and approximately 30% distilled water was incorporated to form a cohesive mass from the powdered feed. The feed was then extruded into strips with a diameter of 2.0 mm using a pellet feed machine (KS-180; Jiangsu Jinggu Rice Mill Co., Ltd., Danyang, China), dried at a constant temperature of 40 °C for 12 h, and stored at −20 °C until used.

### 2.2. Experimental Animals and Conditions

The *H. wyckioides* used in this experiment were provided by the Fish Species Technology Extension Station (Kunming, China) and approved by the Animal Research and Ethics Committee of Yunnan Agricultural University (approval ID: YAUIACUC-2019-A0211; approval date: 11 May 2019). This study was conducted in accordance with the Regulations for the Administration of Affairs Concerning Experimental Animals, as approved by the State Council of the People’s Republic of China on 31 October 1988. The juvenile *H. wyckioides* utilized in this experiment were hatched from the same artificially cultured batch, with an initial average body weight of 3.21 ± 0.01 g. Prior to the commencement of the experiment, the juvenile fish were acclimated to the breeding environment by being raised on commercial feed (TR-2242, EWOS Inc., Puerto Montt, Chilean, Salmofood feed) for 2 weeks. At the onset of the feeding trial, 360 fish were fasted for 24 h and subsequently randomly distributed into 12 culture cages. Each dietary group consisted of three cages, with 30 fish housed in each cage (1.5 m × 0.5 m × 0.6 m). The aquaculture water was aerated and dechlorinated from tap water, while the circulating system utilized both mechanical and biological filtration methods. The water temperature was maintained at 27 ± 1 °C. All the cages were equipped with continuous aeration and were maintained under a natural photoperiod. The fish were hand-fed twice daily (at 07:00 and 17:00) until satiation for 8 weeks. After 30 min of afternoon feeding, any remaining feed and feces were removed by aspiration.

### 2.3. Sample Collection

At the conclusion of the experiment, the fish were sampled following a 24 h fasting period. The fish in each tank were anesthetized using eugenol (1:12,000; Shanghai Reagent Corporation, Shanghai, China). All the fish were counted to assess the survival rate, body weight gain (WG), daily growth coefficient (DGC), feed conversion ratio (FCR), and protein efficiency ratio (PER). Five fish from each cage were randomly selected and stored at −20 °C for proximate composition analysis. Six tubes of tail vein blood samples were collected from six fish in each tank using a heparinized syringe and a 1.5 mL centrifuge tube; these samples were allowed to clot for 4 h at 4 °C. Maintaining the temperature at 4 °C, the blood samples were centrifugated at 3000× *g* for 10 min. Subsequently, the supernatants were collected to obtain plasma samples, which were stored at –80 °C for subsequent analyses. Six fish per tank were then dissected and six separate liver, foregut, and stomach samples were collected in EP tubes for biochemical analysis. Liver and muscle samples were collected from 6 fish per tank and placed in freezing tubes to analyze the relative expression of mRNA. All samples were stored at −80 °C for later analysis.

### 2.4. Analyses

#### 2.4.1. Proximate Composition of Experimental Diet and Fish Carcasses

The proximate composition of both experimental diets and fish carcasses were determined using the methodology outlined by AOAC (version 2000). Moisture content was assessed by drying the samples in an oven at 105 °C until a constant weight was achieved. Crude protein was quantified using the Kjeldahl method (N × 6.25), while crude lipid content was determined by the Soxhlet extraction method. Crude ash was obtained by combustion in a resistance furnace at 550 °C for 16 h. Total energy was measured using a microcomputer automatic bomb calorimeter (ZDHW-5000; Hebi Zhongchuang Instrument Co., Ltd., Hebi, China).

#### 2.4.2. Digestive Enzymes Activities

Foregut samples were prepared according to the methodology described in our previous study [8]. Gut samples were homogenized in ice-cold physiological saline (0.85% *w*/*v*) and subjected to centrifugation at 6000× *g* for 20 min under temperature-controlled conditions. The supernatants obtained were subsequently analyzed for intestinal trypsin (ultraviolet colorimetric method), pepsin (colorimetric method), lipase (methyl resorufin substrate method), and amylase (starch–iodine colorimetric method), were evaluated using commercial kits (Nanjing Jiancheng Bioengineering Institute, Jiangsu, China), following the manufacturer’s instructions.

#### 2.4.3. Plasma Biochemical Parameters

Plasma urea nitrogen (BUN, Urease method), ammonia (NH3-N, protein-free filtrate method), albumin, and malondialdehyde (MDA, TBA method) contents, as well as aspartate aminotransferase (AST, IFCC method), alanine aminotransferase (ALT, IFCC method), γ-glutamyltransferase (γ-GT, GPNA substrate method), and alkaline phosphatase (AKP, microenzyme labeling method) activities were analyzed using commercial kits (Nanjing Jiancheng Bioengineering Institute, Jiangsu, China). Additionally, plasma insulin (INS, enzyme-linked immunosorbent assay), insulin-like growth factor (IGF-1, enzyme-linked immunosorbent assay) and growth hormone (GH, enzyme-linked immunosorbent assay) levels, as well as glutamate dehydrogenase (GDH, enzyme-linked immunosorbent assay) and adenosine monophosphate deaminase (AMPD, enzyme-linked immunosorbent assay) activities, were measured using ELISA kits (R&D Systems, Inc., Minneapolis, MN, USA) in accordance with the manufacturer’s instructions.

#### 2.4.4. Biochemical Parameters in Liver and Muscle

Liver samples were prepared in accordance with our previous study [8]. The hepatic nitric oxide (NO, microplate method) content, as well as catalase (CAT, ammonium molybdate method), AKP, peroxidase (POD, colorimetric method), superoxide dismutase (SOD, hydroxylamine method), lactate dehydrogenase (LDH, microplate method), glutathione peroxidase (GPx, microplate method), glutathione reductase (GR, GRAC method), AST, and ALT activities were measured using commercial kits (Nanjing Jiancheng Bioengineering Institute, Nanjing, China). Additionally, the hepatic γ-GT, GDH, and AMPD activities were quantified using ELISA kits (R&D Systems, Inc., Minneapolis, MN, USA), following the manufacturer’s instructions.

#### 2.4.5. RNA Extraction, cDNA Synthesis, and Real-Time PCR Analysis

The experimental methods for RNA extraction, cDNA synthesis, and real-time PCR analysis were detailed by Zhang et al. (2019) [8]. Total RNA was extracted from liver and muscle tissues using the RNAiso Plus Kit (Takara Bio. Inc., Otsu, Japan) and quantified with a Nanodrop 2000 spectrophotometer (Themo Scientific, Waltham, MA, USA). The integrity of the RNA was assessed via 1% agarose gel electrophoresis. cDNA was synthesized from the extracted RNA using the Thermo Scientific Revert Aid First Strand cDNA Synthesis Kit (Themo Scientific, Waltham, MA, USA) and stored at −20 °C until further use. Specific primers were designed based on the genes amplified and sequenced from fragments of the proximal species available in GenBank. Primers for the target genes, as well as the reference gene (18S rRNA), were also designed based on the GenBank database (Table 2). The *β-actin* housekeeping gene served as an endogenous reference to normalize the template amount for real-time PCR. All primers were synthesized by Sangon Biotech Shanghai Co., Ltd. (Shanghai, China). The reactions were conducted on a real-time quantitative PCR machine using SYBR Green I (Takara Bio. Inc., Otsu, Japan) as the fluorescent dye. Statistical calculations were performed using the 2^−ΔΔCt^ method, with comparative Ct values analyzed to determine the relative expression of these genes.

### 2.5. Statistical Analysis

The normality of the data and homogeneity of variance were assessed prior to analysis. All data were subjected to one-way analysis of variance (ANOVA), with Tukey’s method applied for multiple comparison analysis. Results were presented as mean ± SEM (*n* = 3) with differences considered statistically significant at *p* < 0.05. Statistical analyses were performed using SPSS version 16.0 for Windows.

## 3. Results

### 3.1. Growth Performance

After eight weeks of feeding, all the fish exhibited good health and activity levels. The survival rate was unaffected by the dietary inclusion of RSC (*p* > 0.05; Table 3). Similarly, feed intake and FCR showed no significant differences attributable to dietary RSC inclusion (*p* > 0.05). However, final weight, WG, and DGC decreased with increasing dietary RSC levels, and these were significantly lower in the R45 group compared to the R0 and R15 groups (*p* < 0.05). Additionally, PER was significantly lower in the R30 and R45 groups compared to the R0 and R15 groups (*p* < 0.05).

### 3.2. Digestive Enzyme Activity

The pepsin and intestinal lipase activities were not significantly affected by the dietary inclusion of RSC (*p* > 0.05; Table 4). However, the intestinal trypsin activity was significantly lower in the R30 and R45 groups compared to the R0 and R15 groups (*p* < 0.05). The intestinal amylase activity was significantly lower in the R30 group compared to the R0 and R45 groups (*p* < 0.05).

### 3.3. Antioxidant Parameters in Plasma and Liver

The lowest plasma MDA content was found in the R30 group (*p* < 0.05; Table 5). The activities of plasma AKP as well as hepatic SOD, GR, POD, GPx, and AKP were unaffected by the dietary inclusion of RSC (*p* > 0.05). In contrast, the hepatic CAT activity was significantly lower in the R45 group compared to the R0, R15, and R30 groups (*p* < 0.05). Additionally, the hepatic NO content decreased with the increase in dietary RSC inclusion, and was significantly lower in the R15 and R45 groups compared to the R0 group (*p* < 0.05). Conversely, the hepatic LDH activity was significantly lower in the R15 group compared to the R30 and R45 groups (*p* < 0.05).

### 3.4. Biochemical Parameters in Plasma and Liver

The plasma GDH activity decreased with the increase in dietary RSC inclusion, and this was significantly lower in the R45 group compared to the R15 group (*p* < 0.05; Table 6). Similarly, the plasma AST activity was significantly lower in the R30 and R45 groups compared to the R0 and R15 groups (*p* < 0.05). In contrast, the plasma AMPD activity was enhanced with increasing dietary RSC inclusion, and this was significantly higher in the R30 and R45 groups compared to the R0 and R15 groups (*p* < 0.05). However, the hepatic activities of GDH, AMPD, and ALT, as well as plasma activity of albumin, NH3-N, BUN, insulin, GH, IGF-1, and ALT, were unaffected by dietary RSC inclusion (*p* > 0.05). Additionally, the hepatic GPx activity was significantly lower in the R0 and R15 groups compared to the R30 and R45 groups (*p* < 0.05). The hepatic AST activity was significantly lower in the R45 group compared to the R30 group (*p* < 0.05).

### 3.5. Protein Metabolism-Related Gene Expression Level in Liver and Muscle

**Figure 1:**
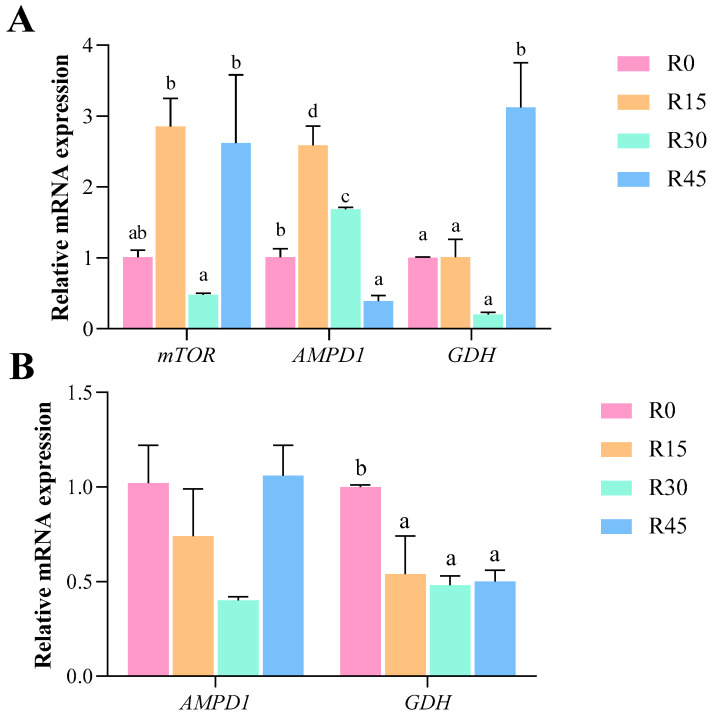
The mRNA expression of protein metabolism-related parameters in liver (**A**) and muscle (**B**) of *H. wyckioides* fed diets with different levels of RSC. Values are means with their standard errors represented by vertical bars (*n* = 6). a,b,c,d bars with different superscripts are significantly different (*p* < 0.05). *mTOR*, mammalian target of rapamycin; *AMPD1*, adenosine monophosphate deaminase 1; *GDH*, glutamate dehydrogenase.

The hepatic mRNA expression level of *AMPD1* initially increased and then declined with increasing dietary RSC inclusion, and this was significantly higher in the R15 and R30 groups compared to the R0 group (*p* < 0.05; Figure 1). In contrast, the hepatic mRNA expression level of *mTOR* first declined and then increased with raising dietary RSC inclusion, and this was significantly lower in the R30 group compared to the R15 and R45 groups (*p* < 0.05). The hepatic mRNA expression level of *GDH* was significantly higher in the R45 group compared to the other groups (*p* < 0.05). However, the hepatic mRNA level of *IGF-1* was unaffected by the dietary inclusion of RSC (*p* > 0.05). Similarly, the muscular mRNA level of *AMPD1* remained unaffected by dietary RSC inclusion (*p* > 0.05). The muscular mRNA level of *GDH* declined with increasing dietary RSC inclusion, and this was significantly lower in the R15, R30, and R45 groups compared to the R0 groups (*p* < 0.05).

### 3.6. Whole-Body Composition

The whole-body moisture, crude protein, crude lipid, and ash contents were not significantly influenced by the dietary inclusion of RSC (*p* > 0.05; Table 7).

## 4. Discussion

In this study, the dietary inclusion of 15% RSC did not adversely affect the growth performance of *H. wyckioides*, while higher substitution levels resulted in negative effects. This finding suggested that the dietary inclusion of 15% RSC was optimal for replacing FM. Prior studies have indicated that feeding Nile tilapia (*Oreochromis niloticus*) a diet containing 30% RSC instead of peanut cake yielded the best specific growth rate, FCR, and PER [24]. Similarly, the complete replacement of soybean protein isolate with rubber seed protein did not adversely affect the feed intake, DGC, PER, and FCR in the omnivorous fish species rohu (*Labeo rohita*) [23]. These findings, in conjunction with the results of the current experiment, indicate that RSC can serve as a potential and viable plant protein source for the diets of omnivorous fish in moderate amounts without negatively impacting growth. However, the present study also demonstrated that the inclusion of 45% RSC reduced the growth performance of *H. wyckioides*. In contrast, another omnivorous freshwater fish species, common carp (*Cyprinus carpio*), exhibited improved growth performance when 20–40% RSC treated with benzene, cold water, and boiling water was included in the diet, compared to untreated RSC [25]. This improvement may be attributed to the reduction in antinutritional factors in the treated RSC, which enhanced feed utilization in fish. Additionally, another by-product of rubber seeds, rubber seed meal (RSM), can be included in the rations of Nile tilapia at a hydrolyzed level of 50% without negatively affecting growth performance [26]. However, rubber seeds have certain disadvantages, including their impact on fish growth due to poor palatability [27]. Furthermore, untreated RSC contains antinutritional factors such as cyanide and phytate phosphorus, which are harmful to fish [28]. The negative impact of high RSC inclusion on the growth of *H. wyckioides* observed in this experiment may also be attributed to these detrimental factors.

The mechanisms through which fish ingest and assimilate nutrients are influenced by various factors. Notably, the activities of digestive enzymes in intestinal epithelial cells play a crucial role in nutrient utilization [29]. The present study observed a decrease in the activities of trypsin and intestinal amylase with the inclusion of RSC in the diet. Similarly, dietary inclusion of RSM inhibited the digestive enzyme activity in tilapia (*Oreochromis niloticus × O. aureus*) [30]. In contrast, a trial involving rohu demonstrated that the activities of trypsin and intestinal amylase remained unchanged when rubber protein isolate was substituted for soybean protein isolate [23]. The discrepancies in these findings may be attributed to species differences and the presence of antinutritional factors. Antinutritional components in rubber seed, such as tannins, phytic acid, and cyanide, may inhibit fish growth by negatively affecting nutrient absorption and digestion [23]. Specifically, tannins have been shown to inhibit intestinal amylase activity [31]. Additionally, phytic acid may limit the absorption of various trace elements and reduce the digestibility of protein and starch within the digestive tract, potentially explaining the inhibition of digestive enzyme activity associated with phytic acid [32]. Furthermore, high levels of cyanogenic glucosides in rubber seed [33], which hydrolyze to produce toxic cyanide, likely contribute to the reduced digestive enzyme activities observed in fish diets with high RSC inclusion in this study. However, not only RSC, but also most plant proteins contain high levels of antinutritional factors, and when added in large quantities they also seriously affect the digestive enzyme activities of fish [34]. Nowadays, several treatments have been realized to reduce the antinutritional factors in rapeseed and soybean meal [35,36], and rubber seeds can be treated in a similar way, eliminating or reducing the antinutritional factors provides a more effective strategy for adding large quantities of RSC to fish feeds. Previous studies have shown that the cyanide content in rubber seed decreased with extended storage time and that treatments such as fermentation, boiling, and defatting can effectively reduce the levels of cyanides and tannins in RSC and its by-products [25,37,38,39].

In our previous investigation, high inclusion levels of dietary RSC resulted in a decrease in the glutamate level within the formulation [30]. Dietary non-essential amino acids, such as glutamate, play a crucial role in preventing the catabolism of essential amino acids in fish, thereby influencing amino acid balance and protein metabolism [40]. This study indicated that RSC had no significant effect on plasma albumin, BUN, and NH3-N levels; however, the inclusion of 45% RSM significantly reduced the plasma and hepatic AST activity. The activities of AST and ALT are critical for nitrogen metabolism and ammonia production [41]. Research on Japanese seabass (*Lateolabrax japonicus*) and rohu have shown that an unbalanced amino acid diet can lead to a decrease in ketoacids, which in turn lower hepatic ALT and AST activities [23,42]. In the present study, an increase in plasma AMPD activity was observed when the diet contained 30% to 45% RSC. Furthermore, the relative expression of hepatic AMPD1 was up-regulated with the inclusion of 15% and 30% RSC in the diet. AMPD is a vital component of the purine nucleotide cycle and plays a significant role in ammonia generation in the skeletal muscles of vertebrates, particularly in fish [43,44]. Consequently, these parameters can be considered important markers or regulators of protein utilization and ammonia excretion, suggesting that the reduced glutamate intake caused by high RSC levels in the diet may stimulate protein degradation and increase ammonia production. Additionally, glutamate serves as a substrate for GDH, which reversibly catalyzes the deamination of glutamate [45,46]. Conversely, γ-GT can catalyze the hydrolysis of glutathione to replenish glutamate and cysteinylglycine in the organism [47]. The observed activation of hepatic γ-GT activity and alterations in hepatic and muscle GDH expression levels in this experiment can be attributed to the decreased intake of glutamate resulting from the replacement of FM with RSC in the feed. In summary, the inclusion of 15% RSC in the feed did not significantly affect protein degradation or amino acid metabolism in fish. However, the incorporation of 45% RSC may result in a reduction in the glutamate level, which could stimulate protein degradation and subsequently impact amino acid metabolism. This decline in growth performance observed in the R45 group may be associated with these effects. From the perspective of protein degradation and amino acid metabolism in fish, high levels of added RSC and high levels of added canola meal had similar results, with protein degradation possibly due to amino acid imbalance occurring despite the same supplementation of essential amino acids [48]. And we believe that RSC can also be treated with soybean meal and canola meal to improve its amino acid balance through fermentation so that it can be added in large quantities.

The antioxidant protection mechanism is a multifaceted system influenced by nutritional and feeding behaviors, among other factors. It consists of both enzymatic and non-enzymatic components [49]. MDA is generated when oxygen radicals attack unsaturated fatty acids in the cell membrane, resulting in cellular damage as these radicals interact with protein-free amino acids, leading to intramolecular and intermolecular protein crosslinking [50]. Antioxidant enzymes, such as SOD and CAT, function as the first line of defense against oxidative stress within cells [30,51]. In the present study, the incorporation of 45% RSC into the diet resulted in a significant increase in plasma MDA level and a notable decrease in hepatic CAT activity. This finding suggests that a high level of replacement of FM with RSC may promote oxidative stress and, to some extent, diminish the antioxidant capacity in *H. wyckioides*. A previous study on Nile tilapia had also shown that elevated level of RSC can reduce antioxidant enzyme activities [30]. Furthermore, LDH is a glycolytic enzyme, where increased LDH activity indicates tissue damage [52]. NO is a critical immunomodulatory factor that regulates the immune system [53]. When 45% of RSC replaced FM in the diet, hepatic LDH activity increased, while hepatic NO level significantly decreased, further suggesting that high RSC levels may induce tissue damage in fish. Non-essential amino acids, such as arginine and glutamic acid, are involved in the regulation of NO synthesis. A decrease in dietary amino acid levels can lead to a reduction in the body’s NO level [54,55]. The results of this experiment indicated that low level of RSC replacement for FM did not adversely affect the immune system; however, high levels of RSC replacement may lead to decrease NO level due to a reduction in glutamate in the diet, ultimately impacting immune function.

## 5. Conclusions

In summary, the inclusion of 15% RSC in diet did not adversely affect the growth, digestive enzyme activity, or antioxidant capacity of *H. wyckioides*. However, the inclusion level of RSC beyond 15% may affect the antioxidant capacity and amino acid metabolism of fish, potentially due to the presence of antinutritional factors and amino acid imbalances in RSC. Therefore, the dietary inclusion of 15% RSC for *H. wyckioides* was recommended.

## Figures and Tables

**Table 1:** Ingredients and proximate composition (% dry matter) of the experimental diets.

Ingredients	R0	R15	R30	R45
Fish meal ^1^	52.00	47.80	43.70	39.50
Rubber seed cake ^1^	0.00	15.00	30.00	45.00
Fish oil ^1^	0.90	0.60	0.30	0.00
Soybean oil ^1^	4.80	3.10	1.40	0.00
DL-Methionine ^3^	0.00	0.07	0.15	0.22
L-Lysine ^3^	0.00	0.15	0.31	0.47
L-Leucine ^3^	0.00	0.07	0.15	0.22
Wheat middling ^1^	25.55	21.46	15.24	10.84
Wheat starch ^1^	13.00	8.00	5.00	0.00
Soybean lecithin ^1^	0.50	0.50	0.50	0.50
Vitamin C ^1^	0.02	0.02	0.02	0.02
Ca(H_2_PO4)_2_^7^	1.20	1.20	1.20	1.20
NaCl ^7^	0.20	0.20	0.20	0.20
Ethoxyquin (30%) ^6^	0.03	0.03	0.03	0.03
Choline chloride (50%) ^6^	0.30	0.30	0.30	0.30
Vitamin mixture ^4^	0.50	0.50	0.50	0.50
Mineral mixture ^5^	0.50	0.50	0.50	0.50
Cr_2_O_3_^7^	0.50	0.50	0.50	0.50
Proximate composition				
Dry matter (DM, %)	90.79	91.20	90.17	89.27
Crude protein (% DM)	40.72	41.08	41.54	40.99
Crude lipid (% DM)	9.88	9.87	10.26	10.28
Nitrogen free extract (% DM)	36.80	35.12	34.05	34.25
Ash (% DM)	11.06	12.01	11.89	11.87
Gross energy (MJ/kg)	20.59	21.18	21.59	22.17

^1^ Supplied by Kunming Tianyuan Feed Co., Ltd. (Yunnan, China); fish meal, 73.6% crude protein, 9.0% crude lipid; rubber seed meal, 30.6% crude protein, 15.8% crude lipid. ^3^ Supplied by Shanghai Hanhong Chemical Co., Ltd. (Shanghai, China). ^4^ Vitamin premix (g/kg mixture): retinyl acetate (2,800,000 IU/g), 2; cholecalciferol, 0.03; DL-α-tocopheryl acetate, 30; menadione, 3; thiamine hydrochloride, 8; riboflavin, 11; pyridoxine hydrochloride, 8; vitamin B12, 0.02; ascorbic acid, 50; folic acid, 1; biotin 0.1; niacin, 30; calcium D-pantothenate, 32; inositol, 25. ^5^ Mineral premix (g/kg mixture): MgSO4•7H2O, 180; KI, 1; FeSO4•H2O, 260; ZnSO4• H2O, 180; CuSO4•5H2O, 25; Na2Se2O3, 0.01; MnSO4•H2O, 180; CoCl2•6H2O, 0.75. ^6^ Yuanyou Biotechnology Co. (Xian, China). ^7^ Sangong Biological Engineering Co Ltd. (Shanghai, China).

**Table 2:** The sequences of primers used in real-time PCR reactions.

Primer	Sequence (5′-3′)	Tm (°C)
*AMPD1*	F: CCACTATTGACCCACAGTCATACC	56.7
	R: TATGCTTGGATTCATCGTCAACAC	
*GDH*	F: TCAAAATCAACCCCAAAAACTTCT	59.6
	R: ATCAGGGGCAGGGACATCAATA	
*IGF-1*	F: GGGGACCGGGGCTTTTATT	60.1
	R: GTGTGCCGTTGCTCTCGTA	
*mTOR*	F: AAGCCGCGTCACATCACACC	59.1
	R: ATCAAAGCGCTCCTCCATCAG	
*β-actin*	F: GGCCGTGACCTGACTGAATACCTC	61.3
	R: AATGCCCATCTCCTGCTCAAAGTC	

*AMPD1*: adenosine monophosphate deaminase 1; *GDH*: glutamate dehydrogenase; *IGF-1*: insulin-like growth factors-1; *mTOR*: mammalian target of rapamycin.

**Table 3:** Growth performance of *H. wyckioides* fed diets with different levels of rubber seed cake.

	R0	R15	R30	R45
Final weight (g)	20.65 ± 0.67 ^b^	21.59 ± 0.29 ^b^	19.12 ± 0.28 ^ab^	17.18 ± 0.86 ^a^
Weight gain	5.50 ± 0.13 ^b^	5.76 ± 0.08 ^b^	5.04 ± 0.09 ^ab^	4.39 ± 0.24 ^a^
DGC (%/day)	2.27 ± 0.08 ^b^	2.34 ± 0.02 ^b^	2.15 ± 0.02 ^ab^	1.98 ± 0.07 ^a^
Feed intake (g/kg MBW/d)	8.26 ± 0.20	8.33 ± 0.14	8.27 ± 0.15	7.93 ± 0.23
Feed conversion rate	0.90 ± 0.02	0.89 ± 0.02	0.94 ± 0.01	0.97 ± 0.03
Protein efficiency ratio	2.73 ± 0.05 ^b^	2.75 ± 0.06 ^b^	2.56 ± 0.02 ^a^	2.52 ± 0.08 ^a^
Survival (%)	98.89 ± 1.11	97.78 ± 1.11	96.67 ± 3.33	93.33 ± 3.84

Values are presented as means ± SEM (*n* = 3), and the means in the same row with different superscript letters are significantly different from each other (*p* < 0.05). DGC, daily growth coefficient. Note: Survival rate (SR, %) = Sf/Si × 100, weight gain (WG) = (FBW − IBW)/IBW, daily growth coefficient (DGC, %/d) = (FBW1/3 − IBW1/3)/d × 100, mean metabolic body weight (MBW) = [(IBW/1000)0.75 + (FBW/1000)0.75]/2, total feed intake (g/fish) = dry diet intake/total number of fish, feed intake (g/kg MBW per day) = DDI/MBW/d, feed conversion rate (FCR) = DDI/(FBW − IBW), protein efficiency ratio (PER) = (FBW − IBW) / protein intake; where IBW, FBW, and DDI are the initial body weights (g), final body weights (g), and dry diet intake per fish (g DM/fish), respectively; d is feeding days; Si and Sf are the numbers of surviving fish at initial and final stages of the test.

**Table 4:** Digestive enzyme activities in the gastrointestinal tract of *H. wyckioides* fed diets with different levels of rubber seed cake.

	R0	R15	R30	R45
Stomach				
Pepsin (U/mg protein)	15.67 ± 0.61	15.94 ± 2.31	16.12 ± 2.44	14.81 ± 0.47
Foregut				
Trypsin (U/µg protein)	1.38 ± 0.06 ^b^	1.10 ± 0.13 ^ab^	0.83 ± 0.06 ^a^	0.94 ± 0.07 ^a^
Lipase (U/g protein)	32.31 ± 6.49	34.39 ± 1.39	19.19 ± 2.52	28.63 ± 5.17
Amylase (U/mg protein)	2.70 ± 0.27 ^b^	2.31 ± 0.22 ^ab^	1.37 ± 0.27 ^a^	2.74 ± 0.30 ^b^

Values are presented as means ± SEM (*n* = 3), and the means in the same row with different superscript letters are significantly different from each other (*p* < 0.05).

**Table 5:** Antioxidant-related parameters in plasma and liver of *H. wyckioides* fed diets with different levels of rubber seed cake.

	R0	R15	R30	R45
Plasma				
AKP (U/dL)	15.18 ± 2.41	22.12 ± 2.14	11.35 ± 3.91	12.71 ± 2.57
MDA (mmol/L)	7.21 ± 0.82 ^ab^	6.90 ± 1.22 ^ab^	4.65 ± 1.05 ^a^	13.02 ± 2.33 ^b^
Liver				
SOD (U/mg protein)	34.46 ± 0.73	29.89 ± 2.40	33.73 ± 1.09	34.52 ± 1.13
CAT (U/mg protein)	10.46 ± 0.30 ^b^	11.05 ± 0.72 ^b^	11.85 ± 0.73 ^b^	7.94 ± 0.95 ^a^
POD (U/mg protein)	1.17 ± 0.14	0.89 ± 0.05	0.93 ± 0.04	0.98 ± 0.07
GPx (U/µg protein)	0.14 ± 0.01	0.12 ± 0.01	0.14 ± 0.01	0.13 ± 0.01
GR (U/g protein)	7.99 ± 0.85	6.67 ± 1.02	7.57 ± 0.83	5.75 ± 0.72
LDH (U/g protein)	4.07 ± 0.12 ^ab^	3.49 ± 0.20 ^a^	5.12 ± 0.25 ^c^	4.55 ± 0.25 ^bc^
NO (µmol/g protein)	6.64 ± 0.40 ^b^	4.19 ± 0.20 ^a^	4.92 ± 0.69 ^ab^	4.18 ± 0.20 ^a^
AKP (U/g protein)	12.20 ± 1.57	9.20 ± 1.07	9.44 ± 0.48	9.40 ± 1.46

Values are presented as means ± SEM (*n* = 3), and the means in the same row with different superscript letters are significantly different from each other (*p* < 0.05). AKP, alkaline phosphatase; MDA, malondialdehyde; SOD, superoxide dismutase; CAT, catalase; POD, peroxidase; GPx, glutathione peroxidase; GR, glutathione reductase; LDH, lactate dehydrogenase; NO, nitric oxide.

**Table 6:** Protein metabolism-related parameters in plasma and liver of *H. wyckioides* fed diets with different levels of rubber seed cake.

	R0	R15	R30	R45
Plasma				
Albumin (g/L)	15.34 ± 2.19	15.34 ± 0.57	10.85 ± 1.56	11.35 ± 1.27
BUN (mmol/L)	9.86 ± 1.01	10.00 ± 0.78	8.40 ± 0.95	9.27 ± 0.67
Insulin (mU/L)	8.11 ± 0.45	8.20 ± 0.27	7.96 ± 0.30	7.64 ± 0.17
GH (µg/L)	17.60 ± 0.31	17.51 ± 0.30	17.40 ± 0.08	17.26 ± 0.18
IGF-1 (µg/L)	19.90 ± 1.28	16.86 ± 0.83	19.52 ± 0.80	17.37 ± 0.46
AMPD (U/L)	14.05 ± 0.23 ^a^	15.08 ± 0.71 ^a^	21.22 ± 0.39 ^b^	19.60 ± 0.95 ^b^
GDH (U/L)	9.63 ± 0.42 ^ab^	10.49 ± 0.46 ^b^	9.19 ± 0.50 ^ab^	8.42 ± 0.34 ^a^
AST (U/L)	29.21 ± 0.33 ^b^	30.00 ± 1.17 ^b^	20.43 ± 1.69 ^a^	21.87 ± 0.77 ^a^
ALT (U/L)	10.02 ± 1.73	9.84 ± 1.34	8.52 ± 0.96	6.64 ± 0.40
Liver				
AMPD (U/g protein)	0.69 ± 0.03	0.66 ± 0.01	0.78 ± 0.05	0.67 ± 0.03
GDH (U/g protein)	0.17 ± 0.00	0.16 ± 0.00	0.18 ± 0.01	0.17 ± 0.01
AST (U/g protein)	11.00 ± 1.20 ^ab^	10.71 ± 1.38 ^ab^	13.95 ± 1.32 ^b^	7.64 ± 0.48 ^a^
ALT (U/g protein)	15.28 ± 1.36	10.76 ± 0.62	11.60 ± 1.98	9.92 ± 1.17
γ-GT (U/g protein)	0.17 ± 0.01 ^a^	0.26 ± 0.04 ^a^	0.93 ± 0.05 ^b^	1.08 ± 0.09 ^b^

Values are presented as means ± SEM (*n* = 3), and the means in the same row with different superscript letters are significantly different from each other (*p* < 0.05). BUN, blood urea nitrogen; GH, growth hormone; IGF-1, insulin-like growth factors-1; AMPD, adenosine monophosphate deaminase; GDH, glutamate dehydrogenase; AST, aspartate aminotransferase; ALT, alanine aminotransferase; γ-GT, γ-glutamyl transpeptidase.

**Table 7:** The whole-body composition of *H. wyckioides* fed diets with different levels of rubber seed cake.

	Initial	R0	R15	R30	R45
Moisture (%)	84.70	69.77 ± 0.20	69.93 ± 0.16	69.60 ± 0.12	70.48 ± 0.22
Crude protein (%)	10.06	14.90 ± 0.09	15.32 ± 0.06	15.16 ± 0.13	15.16 ± 0.13
Crude lipid (%)	3.80	10.75 ± 0.06	10.31 ± 0.08	11.12 ± 0.10	11.12 ± 0.10
Ash (%)	1.49	3.04 ± 0.04	2.99 ± 0.06	2.56 ± 0.07	2.99 ± 0.06

Values are presented as means ± SEM (*n* = 3).

## Data Availability

The data that support the findings of this study are available on request from the corresponding author. The data are not publicly available due to privacy or ethical restrictions.
